# A novel basolateral type IV secretion model for the CagA oncoprotein of *Helicobacter pylori*

**DOI:** 10.15698/mic2018.01.611

**Published:** 2017-12-09

**Authors:** Silja Wessler, Steffen Backert

**Affiliations:** 1 Dept. of Molecular Biology, University of Salzburg, Austria.; 2 Dept. of Biology, Division of Microbiology, University of Erlangen-Nuremberg, Germany.

**Keywords:** E-cadherin, focal adhesions, Helicobacter, protease, HtrA, CagA, T4SS, claudin, occludin, integrin

## Abstract

Intercellular junctions are crucial structural elements for the formation and maintenance of epithelial barrier functions to control homeostasis or protect against intruding pathogens in humans. Alterations in these complexes represent key events in the development and progression of numerous cancers as well as multiple infectious diseases. Many bacterial pathogens harbor type IV secretion systems (T4SSs), which translocate virulence factors into host cells to hijack cellular processes. The pathology of the gastric pathogen and type-I carcinogen *Helicobacter pylori *strongly depends on a T4SS encoded by the* cag* pathogenicity island (*cag*PAI). This T4SS forms a needle-like pilus and its activity is accomplished by the pilus-associated factors CagL, CagI and CagY which target the host integrin-β_1_ receptor followed by injection of the CagA oncoprotein into non-polarized AGS gastric epithelial cells. The finding of a T4SS receptor, however, suggested the presence of a sophisticated control mechanism for the injection of CagA. In fact, integrins constitute a group of basolateral receptors, which are normally absent at apical surfaces of the polarized epithelium *in vivo*. Our new results demonstrate that T4SS-pilus formation during *H. pylori *infection of polarized epithelial cells occurs preferentially at basolateral sites, and not at apical membranes (Tegtmeyer *et al*., 2017). We propose a stepwise process how *H. pylori* interacts with components of intercellular tight junctions (TJs) and adherens junctions (AJs), followed by contacting integrin-based focal adhesions to disrupt and transform the epithelial cell layer in the human stomach. The possible impact of this novel signaling cascade on pathogenesis during infection is reviewed.

The mechanism of CagA translocation into polarized gastric epithelial cells remained mysterious for a long time. How can *H. pylori* establish contact with integrin-β_1_ at basolateral surfaces of polarized cells to translocate CagA and initiate signaling when the apical and lateral junctions are still intact? Are there other bacterial factors involved in manipulating cell-to-cell junctions during infection, allowing *H. pylori* to invade the intercellular space and approach basal membranes? To achieve this goal, *H. pylori *secretes the serine protease HtrA *in vitro *and* in vivo*, which opens the cell-to-cell junctions through cleavage of crucial junctional proteins. As (*htrA *gene knockout mutants are not available for *H. pylori*, we developed a genetic strategy to induce the expression of an HtrA peptide inhibitor within the bacteria. Using this methodology, we could confirm that proteolytic HtrA activity is required for opening cell-to-cell junctions and paracellular transmigration of *H. pylori* across the polarized epithelial monolayer. In this way,* H. pylori* can target basolateral integrins and trigger CagA delivery.

Transmission electron microscopy (EM) and anti-HtrA immunogold staining was performed on 20 gastric biopsies from *H. pylori*-positive patients. We confirmed that HtrA was secreted *in vivo.* HtrA was also found at apical junctional complexes and in deep intercellular clefts of the damaged gastric epithelium. As the expression of the AJ protein E-cadherin is commonly downregulated in gastric cancer patients, we employed immunohistochemistry to detect the ectodomain of E-cadherin. Compared to *H. pylori*-negative atrophic gastritis biopsies with intestinal metaplasia, we observed a significant downregulation by about 40% of the E-cadherin pattern in *H. pylori*-positive gastric tissues from patients with neoplasia, confirming our previous* in vitro* data. Thus, the large extent of E-cadherin reduction correlated with elevated levels of secreted HtrA and reflects a severe disruption of the mucosal barrier in the *H. pylori*-infected stomach. In the following experiments, we investigated the interaction of *H. pylori *with cultured polarized epithelial cell lines (MDCK, MKN28 and NCI-N87) by scanning EM. About 4 and 8 hours post-infection, *H. pylori* attached to the apical surface of the epithelium, in close proximity to the cell-to-cell junctions. This was accompanied by specific HtrA-mediated cleavage of the TJ proteins occludin and claudin-8, which represent new HtrA substrates, to loosen the epithelium. In agreement with this observation, the bacteria were found both at the apical and basolateral surfaces of the polarized epithelium at 24 hours post-infection. This indicates that *H. pylori *disturbs both the TJs and AJs, and can efficiently enter cell monolayers.

We next closely inspected the T4SS-pilus formation by scanning EM. Remarkably, T4SS-pili were specifically produced by transmigrated basolateral *H. pylori*, while they were nearly absent in apically bound bacteria*.* This implied that T4SS activation appeared predominantly at the basolateral surface of the epithelium. To test this intriguing idea and to approve that bacterial transmigration was necessary for the function of the *H. pylori* T4SS, we analyzed the delivery of CagA, which is tyrosine-phosphorylated by host kinases c-Abl and c-Src upon injection. Using phospho-CagA antibodies and confocal laser scanning microscopy, we demonstrated that phosphorylated CagA co-localized with basolateral integrin-β_1_, thus confirming that the T4SS was activated and translocated CagA into host cells. To validate these findings further, we aimed to suppress HtrA by the E-cadherin-derived peptide inhibitor P1. For this purpose, we placed the P1 sequence (TGTLLLILSDVNDNAPIPEPR) under the control of the arabinose-inducible pBAD system in *H. pylori*. Transmigration assays, transmission EM and Western blotting revealed that dose-dependent induction of P1 expression inhibited HtrA activity, followed by a decrease of bacterial transmigration, as well as CagA translocation and phosphorylation. These findings confirm a model in which the proteolytic activity of HtrA is required for effective bacterial transmigration and injection of CagA into polarized epithelial cells.

To corroborate these findings in more detail, we compared CagA translocation in epithelial cells with the same genetic background, but exhibiting differences in polarization status. Non-polarized AGS cells are commercially available, and do not express E-cadherin. Stable introduction of the *E-cadherin* gene in these cells produced an increase in polarization. Infection of the E-cadherin-deficient AGS cells with P1-expressing *H. pylori *in the presence of arabinose led to efficient CagA translocation and phosphorylation in a time course, but not in E-cadherin-expressing AGS cells. As control, CagA can be similarly well phosphorylated in both cell lines in the absence of arabinose, but with a significant delay in E-cadherin-expressing cells, as expected. Based on these functional experiments, it was elegantly demonstrated that HtrA activity is necessary for injection of CagA during *H. pylori *infection of E-cadherin-expressing polarized AGS cells, but not in non-polarized control cells. Taken together, these data support the view that the *H. pylori* T4SS and HtrA work hand in hand during infection of the polarized epithelium, resulting in basolateral injection and phosphorylation of CagA.

Altogether, we report here on a novel mechanism of integrin receptor-dependent T4SS activation during infection of polarized gastric epithelial cells (Figure 1A). We identified *H. pylori *HtrA as a secreted serine protease *in vitro *and* in vivo* that directly cleaves the TJ factors claudin-8 and occludin as well as the AJ protein E-cadherin in the polarized gastric epithelium (Figure 1B and C). These observations are in agreement with reports on infected patients displaying significant higher serum titers of soluble E-cadherin compared to uninfected control patients. In addition to the properties of HtrA as demonstrated in the present report, changing methylation patterns or accumulation of mutations in the *E-cadherin* gene can further promote the inactivation of E-cadherin in gastric adenocarcinomas. Given that TJs and AJs are crucially important for the epithelial architecture, HtrA-triggered cleavage of junctional factors will ultimately lead to disintegration of cell-to-cell adhesion and disrupt epithelial barrier properties. This can also support the incidence of deep epithelial clefts in *H. pylori*-positive gastric biopsies *in vivo *as described above. Consequently, *H. pylori *can then efficiently move into the intercellular space between neighbouring cells permitting bacterial travel to basolateral locations, T4SS formation and CagA delivery across integrin-β_1_ (Figure 1).

**Figure 1:**
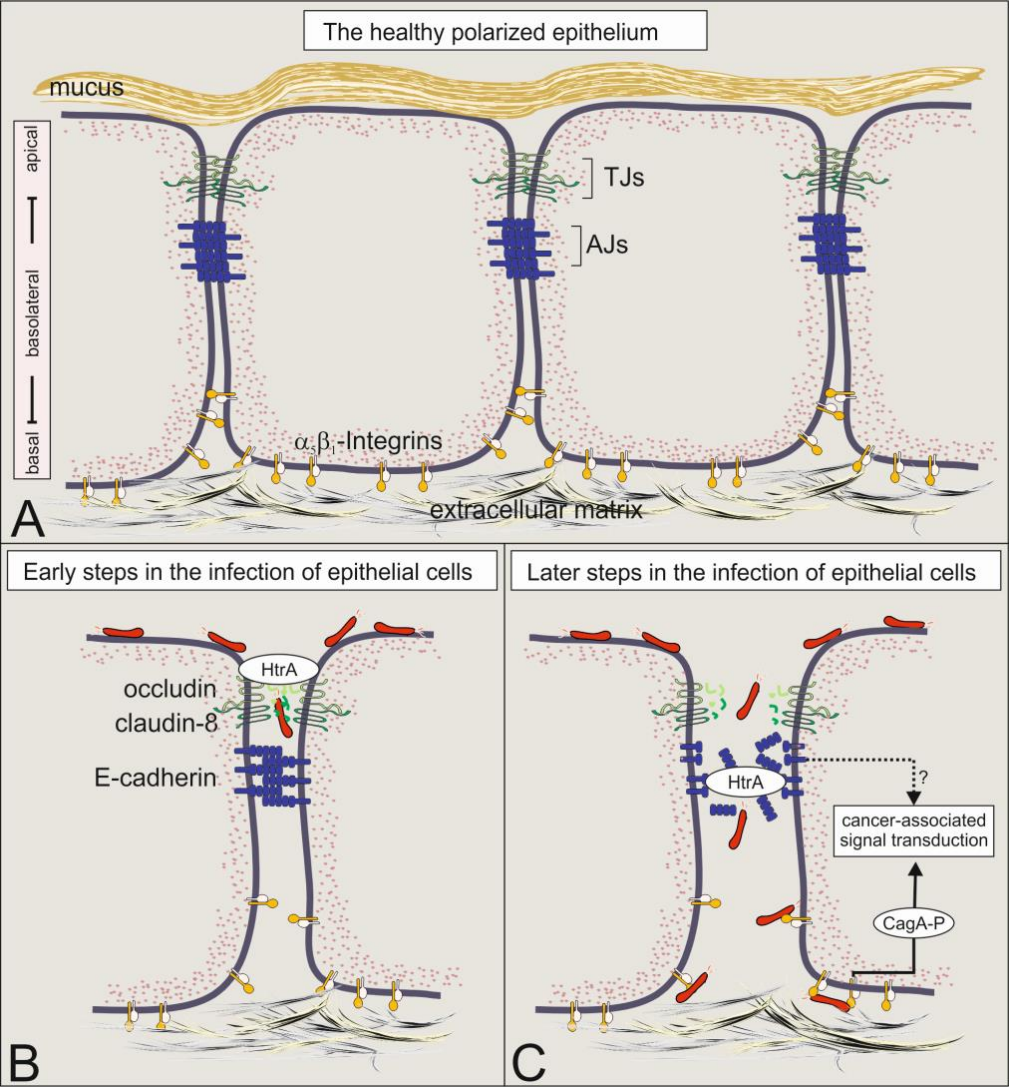
FIGURE 1: Translocation and phosphorylation of CagA in polarized cells require HtrA protease activity. (**A**) A model of the healthy polarized gastric epithelium. The surface of the stomach is protected by the gastric mucus. Polarization of the underlying epithelial cells is mainly established by intercellular adhesion complexes, including tight junctions (TJs) and adherence junctions (AJs). The TJs are directly located above AJs and mark the transition from the apical domain to the basolateral domain. The expression of the T4SS receptor integrin-β_1_ occurs at basolateral and basal domains of polarized epithelial cells and is crucially important for the adherence of cells to the extracellular matrix. (**B**) The current data indicate that *H. pylori* uses a paracellular transmigration route to reach integrin-β_1_ at basolateral surfaces as indicated. For this purpose, secreted HtrA targets specific host cell factors in the TJs (occludin and claudin-8) at early phases of infection. (**C**) At later phases of infection, E-cadherin-based AJs are disrupted, allowing the contact between the T4SS and integrin-β_1_ and subsequently the delivery of CagA into the cytoplasm of host cells. Upon CagA translocation, CagA is tyrosine-phosphorylated and hijacks host cell signaling cascades which are implicated in gastric carcinogenesis.

It is well-established that translocated CagA can bind to a set of more than 24 host cell signaling factors in a phosphorylation-dependent and phosphorylation-independent fashion. In this way, *H. pylori *can manipulate fundamental processes in the gastric epithelium involving cell adhesion, proliferation and cytoskeletal reorganization. Among the events are various cancer-associated signal transduction routes, including Par1b-mediated loss of cell polarity, β-catenin activation and Snail-triggered epithelial-mesenchymal transition. Generally, HtrA-dependent opening of cell-to-cell junctions and T4SS activation highlight an innovative new strategy allowing *H. pylori* persistence and pathogenicity. However, we are still just at the beginning to recognize the impact of *H. pylori* HtrA on cell-to-cell junctions. It is becoming evident that *H. pylori* merges numerous strategies to alter intercellular adhesion, implying multi-step processes in changing cell polarity. In fact, CagA is a highly potent and multifunctional signaling factor, but further studies are needed to acquire deeper insights in the signal transduction complex of CagA. Even though CagA is critical for triggering intracellular signal transmission pathways, work on additional T4SS-associated factors such as CagL, CagY, HopQ, sugar heptose-1,7-bisphosphate (HBP) and many others will ultimately enhance our general understanding and add new facets to our multi-step model of gastric pathogenesis. Since *H. pylori*-induced cell migration has been also reported and strongly contributes to inflammation, wound healing, angiogenesis, invasive growth and metastasis *in vivo*, accumulating information on the mechanisms of modifying cell adhesion and other bacterial factors involved in these activities is very important and might help to improve novel therapeutic intervention schemes for treating gastric diseases in future.

